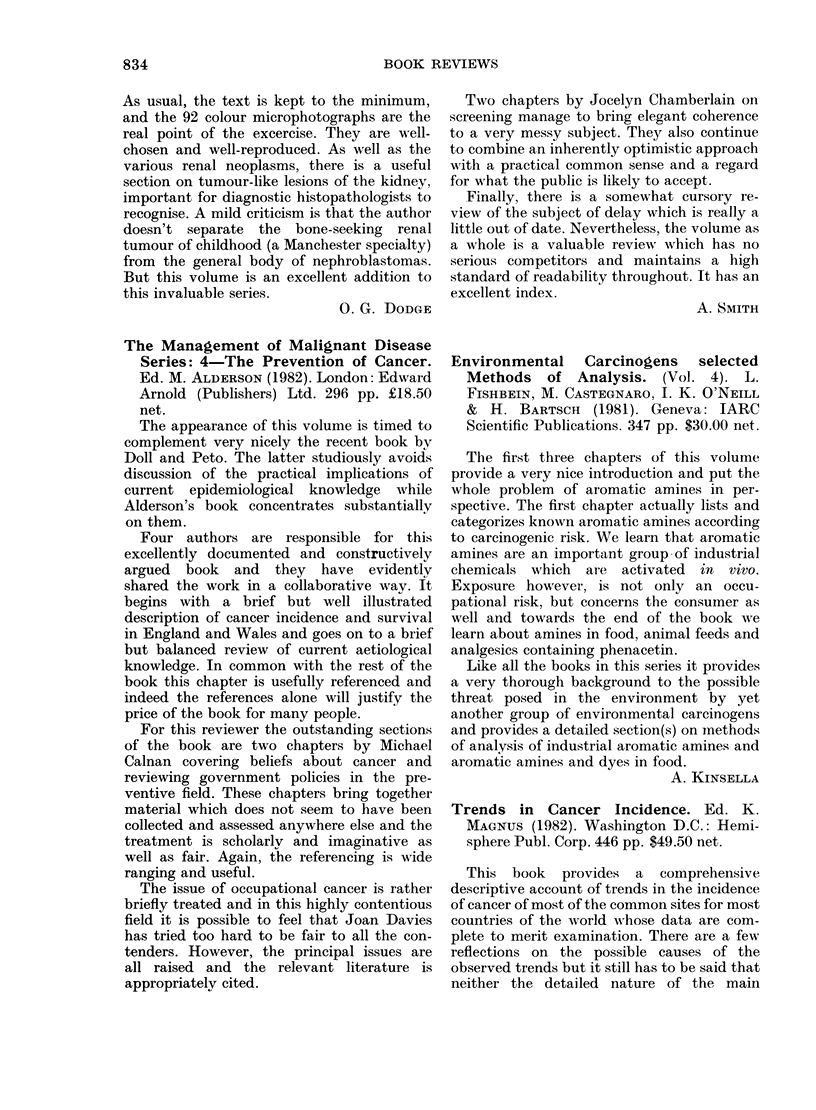# Environmental Carcinogens selected Methods of Analysis

**Published:** 1982-11

**Authors:** A. Kinsella


**Environmental Carcinogens selected Methods of Analysis.** (Vol. 4). L. FISHBEIN, M. CASTEGNARO, I. K. O'NEILL & H. BARTSCH (1981). Geneva: IARC Scientific Publications. 347 pp. $30.00 net.

The first three chapters of this volume provide a very nice introduction and put the whole problem of aromatic amines in perspective. The first chapter actually lists and categorizes known aromatic amines according to carcinogenic risk. We learn that aromatic amines are an important group of industrial chemicals which are activated *in vivo*. Exposure however, is not only an occupational risk, but concerns the consumer as well and towards the end of the book we learn about amines in food, animal feeds and analgesics containing phenacetin.

Like all the books in this series it provides a very thorough background to the possible threat posed in the environment by yet another group of environmental carcinogens and provides a detailed section(s) on methods of analysis of industrial aromatic amines and aromatic amines and dyes in food.

A. KINSELLA